# Effects of conjugated linoleic acid/n-3 and resistance training on muscle quality and expression of atrophy-related ubiquitin ligases in middle-aged mice with high-fat diet-induced obesity

**DOI:** 10.20463/jenb.2017.0028

**Published:** 2017-09-30

**Authors:** Seung-Lyul Oh, Sang-Rok Lee, Jeong-Su Kim

**Affiliations:** 1. Aging & Mobility Biophysics Lab, Dept. of Rehabilitation Medicine, Seoul National University Bundang Hospital, Sungnam Republic of Korea; 2. Institute on Aging, Seoul National University, Seoul Republic of Korea; 3. Department of Kinesiology and Dance, New Mexico State University, NM United States USA; 4. Department of Nutrition, Food and Exercise Science, Florida State University, FL United States USA

**Keywords:** Conjugated linoleic acid (CLA), Omega-3 polyunsaturated fatty acid (n-3 PUFAs), Resistance training, Muscle quality, Muscle atrophy

## Abstract

**[Purpose]:**

To investigate the effects of conjugated linoleic acid (CLA)/n-3 supplements and resistance exercise training (RT) for 20 weeks on muscle quality and genes related to protein synthesis/degradation in middle-aged mice with high-fat diet (HFD)-induced obesity.

**[Methods]:**

Nine-month-old C57BL/6 male mice were randomly assigned to five groups: 1) normal diet (C), 2) high-fat diet (H), 3) H+RT (HRT), 4) H+CLA/n-3 (H-CN), and 5) H+RT+CLA/n-3 (H-RTCN). HFD groups were given a diet containing 60% fat for 20 weeks, and exercised groups underwent progressive RT using weighted ladder climbing. The CLA/n-3 mixed diet contained 1% CLA and 1% n-3. Grip strength was assessed, and triceps were removed. RT-PCR was used to analyze transcript levels.

**[Results]:**

Grip strength of the H group was significantly lower than that of the C group; however, those in the H-CN, H-RT, and H-RTN groups were significantly greater than that in the H group. However, the muscle quality was significantly greater only in the H-RT group compared with the H and H-CN groups. Akt expression decreased in the H-CN, H-RT, and H-RTCN groups compared with those in the C and H groups, whereas mammalian target of rapamycin expression increased in the H, H-CN, H-RT, and H-RTCN groups compared with that in the C group. However, atrogin1 was significantly downregulated in the H-RTCN group compared with that in the H and H-CN groups, and MuRF1 expression was also decreased in the H-RT and H-RTCN groups. Interestingly, atrogin1 and MuRF1 were downregulated in the H-RTCN group compared with that in the H-CN group.

**[Conclusion]:**

HFD-mediated gene expression involved in protein degradation was attenuated following 20-week RT with CLA/n-3. Furthermore, RT with or without CLA/n-3 improved grip strength and muscle quality in middle-aged mice during HFD. Therefore, RT with CLA/n-3 during HFD may improve muscle strength and quality by suppressing protein degradation.

## INTRODUCTION

Obesity is a serious health problem that can be regulated through various approaches, such as scientific, pharmacological, and nutritional/exercise interventions, as well as improvement of lifestyle. Obesity induces negative regulation of genes related to protein synthesis/degradation in skeletal muscle tissue, consistent with the observation of decreased lean body mass and increased fat mass in the aged population. Sarcopenic obesity refers to a new trend in aged individuals who simultaneously demonstrate reductions and increases in lean mass and fat mass, respectively, leading to poor quality of life. High density energy intake (i.e., a high-fat diet) with limited physical activity (i.e., a sedentary life style) induces negative alterations in body composition, possibly leading to early onset of the sarcopenic process.

Maintaining muscle mass is a balance between protein synthesis and protein degradation systems, involving molecular mechanisms that regulate muscle growth and atrophy^1^. There are several signaling pathways that regulate muscle protein synthesis/degradation. Insulin-like growth factor 1 (IGF1), a major activator of muscle hypertrophy, induces an increase in muscle mass by the phosphatidylinositol 3-kinase (PI3K)/Akt/mammalian target of rapamycin (mTOR) signaling pathway, resulting in the activation of downstream effector proteins^2, 3^. When IGF1 binds to its receptor, insulin receptor substrate-1 (IRS-1) is activated, followed by PI3K activation through conversion of intramembranous phosphoinositide-(4,5)-biphosphate to phosphoinositide-(3,4,5)-triphosphate (PIP3)^4^. Subsequently, Akt binds PIP3 and is activated by phosphoinositide-dependent kinase-1 (PDK1). Akt is then released into the cytosol to activate mTOR through PDK1. Akt plays an important role in skeletal muscle hypertrophy via activation of mTOR and glycogen synthase kinase 3β^5^. In contrast, muscle atrophy is associated with an increase in several proteins that promote proteolysis, such as atrogin-1 and Murf-1, while increasing the degradation of myofibrillar protein^6^. Additionally, inhibition of the PI3K/Akt/mTOR signaling pathway is involved in the synthesis of related proteins and accompanied by suppression of muscle protein synthesis^7, 8^.

In many studies, long-term regular exercise interventions are considered an important tool for the prevention and treatment of obesity. In particular, resistance exercise training (RT) is an effective intervention to combat sarcopenia by enhancing myofiber hypertrophy and improving muscle strength, power, and mobility^9^. Muscle quality, referred to as specific tension, is a measure of the strength per unit of muscle mass^10^ and may be a better indicator of muscle function than strength alone^11^.

Conjugated linoleic acid (CLA) and omega-3 polyunsaturated fatty acids (n-3 PUFAs) have received much attention for their anti-obesity effects^12-15^. Eicosapentaenoic acid (EPA) and docosahexaenoic acid (DHA) in long-chain omega-3 fatty acids are major factors affecting metabolism and inflammation. In particular, EPA has been shown to reduce triglycerides and improve blood lipid profiles^17^, and DHA has been reported to enhance lipid oxidation and insulin sensitivity through activation of AMP-activated protein kinase signaling in skeletal muscle^15^. In addition, many studies have reported that CLA increases appetite suppression and fatty acid oxidation^16, 18-20^. Specifically, in animal models of obesity induced by a high-fat diet (HFD), CLA treatment not only compensates for body weight gain, but also increases fatty acid oxidation with a decrease in blood insulin concentrations^19^. Similarly, many studies have reported that CLA treatment induces changes in body composition, decreasing body weight and body fat and increasing lean body mass^20-25^. However, the trans-10 and cis-12 isomers of CLA induce liver hypertrophy and steatosis, and studies have shown that insulin sensitivity is reduced^26-29^. In contrast, n-3 has been reported to prevent obesity and improve insulin sensitivity^30^. In fact, studies have shown that n-3 intake counteracts the side effects of CLA treatment^31^. Intake of CLA and n-3 also enhances the benefits induced by RT, including increased skeletal muscle mass^32^ and muscle strength^33^, ^34^. However, Lee and colleagues^35^ reported that CLA plus n-3 intake did not prevent the reduction in muscle strength and mass due to long-term HFD.

Although CLA and n-3 have attracted interest because of their health-enhancing benefits, few studies have examined muscle quality and gene expression related to protein synthesis and degradation in skeletal muscle with RT in middle-aged mice with HFD-induced obesity. Additionally, their efficacy in attenuating HFD-induced impairments in skeletal muscle remains poorly understood. Therefore, the purpose of this study was to investigate the effects of CLA/n-3 supplements and RT for 20 weeks on muscle quality and genes related to protein synthesis/degradation in middle-aged mice with HFD-induced obesity.

## METHODS

### Animals and experimental design

**Figure 1.**
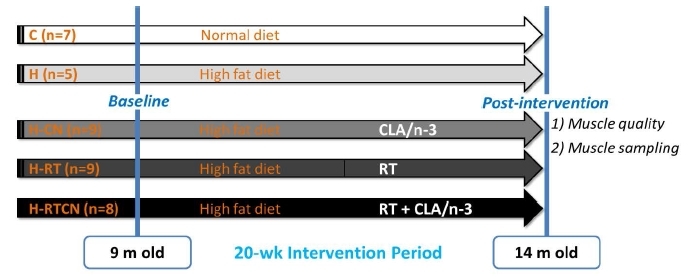
Overall Research Design.

Nine-month-old C57BL/6 male mice were randomly assigned to one of five groups: 1) normal diet control (C, n = 7), 2) HFD control (H, n = 5), 3) HFD with CLA/n-3 (H-CN, n = 9), 4) HFD with RT (H-RT, n = 9), and 5) HFD with RT and CLA/n-3 (H-RTCN, n = 8). All groups except the C group were fed an HFD for 20 weeks. Each group received an HFD alone (H), HFD with CLA/n-3 (H-CN), HFD with RT (H-RT), or HFD with RT and CLA/n-3 combined treatment (H-RTCN) for 20 weeks (Fig. 1). The animals were housed individually in stainless steel cages maintained at 22–24°C with a 12:12 h light:dark cycle. After consumption of the specific diet for 5 months, animals were sacrificed, and muscles were harvested. All procedures were approved by the institutional Animal Care and Use Committee of Florida State University.

**Table 1.** The results of body weight, triceps weight, grip strength, and muscle quality.

	C (n=7)	H (n=5)	H-CN (n=9)	H-RT (n=9)	H-RTCN (n=8)	F
Pre BW, g	32.23±0.75	30.00±1.06	30.98±0.72	31.23±0.73	31.51±0.87	0.794
Post BW, g	29.84±0.77	46.94±1.01^###^	33.19±2.16^†††^	42.27±1.42^###‡‡‡^	38.00±1.77^##††‡^	14.500***
Pre Fat mass, g	14.14±0.86	12.00±1.52	14.00±0.67	13.56±1.00	14.00±1.35	0.509
Post Fat mass, g	15.86±1.26	30.40±1.63^###^	17.44±1.14^†††^	32.22±1.80^###‡‡‡^	19.63±1.13^†††¥¥¥^	28.002***
Triceps/BW, mg/g	35.73±1.59	24.51±2.58^###^	31.25±1.34^#††¥¥^	25.28±1.05^###‡‡^	33.66±1.56^††¥¥¥^	9.906***
Grip Strength/ BW, g/g	48.34±1.13	27.49±1.10^###^	39.24±2.87^#††^	39.64±2.14^#††^	47.26±3.64^†††‡¥^	21.075***
Muscle quality, g/mg	136.52±5.23	119.38±17.25	125.44±6.45	158.99±11.10^†‡^	140.98±9.69	2.439

Values are mean ± standard errors. C, normal diet control; H, high fat diet; H-CN, high fat diet with CLA/n-3; H-RT, high fat diet with resistance training; H-RTCN, high fat diet with CLA/n-3 supplement and resistance training.

*p<.05, significant differences among groups

#p<.05, significantly different from C;

†p<.05, significantly different from H

‡p<.05, significantly different from H-CN

¥p<.05, significantly different from H-RT

**Figure 2.**
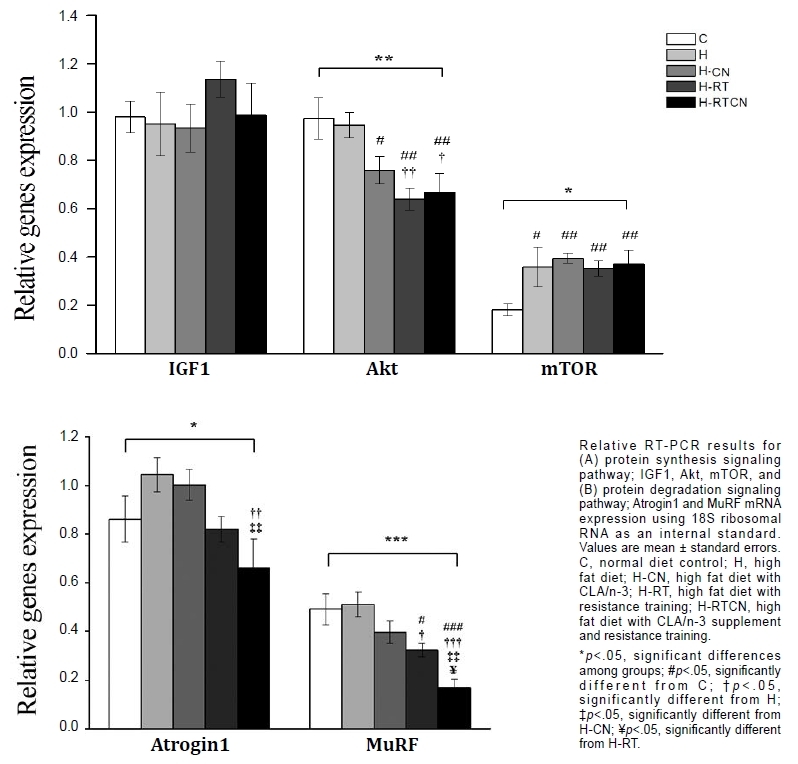
Relative RT-PCR results for genes expression related to protein synthesis/degradation using 18S ribosomal RNA as an internal standard.

### RT

The mice in the exercise groups (H-RT and H-RTCN) were trained to climb a 1-m vertical ladder with an 85° incline three times per week for 20 weeks. During the first week, they were familiarized with climbing up to the top of the ladder with and without weight on their tails. Training sessions were commenced with intensity at 50% body weight (about 15 g) and increased by 1% body weight on a biweekly basis. They performed four sets of three reps (about 38 contractions) with 1-min rest intervals between repetitions and 2-min intervals between sets, 3 days per week for 20 weeks, as described in our previous studies^35-37^.

### CLA/n-3 mix intake

The C group was fed a normal diet (10% fat by energy, 3.8 kcal/g), whereas the HFD groups were given 60% fat (5.2 kcal/g) for 20 weeks, as previously described^35-37^. The CLA/n-3 mixed diet contained 1% CLA (50:50 mix of cis-9, trans-11:trans-10, cis-12) and 1% PUFAs enriched in n-3 fatty acids. Fresh diet was prepared weekly, stored in sealed plastic bags at -20°C to minimize oxidation, and provided daily ad libitum.

### Muscle strength and quality

Muscle strength was measured using a grip strength meter for animals (DFS-101; AMETEK, CA, USA) as previously described^35-37^. All mice adapted with dual grip bars connected to separate strain gauges to allow separate measurements of force with the forelimbs. For each trial, the mice were placed on the dual grip bar and then pulled gently by the scruff of the neck and base of the tail, in a rearward direction, away from the bar. The applied force at the point at which the mouse released its grip for each paw was recorded by two separate strain gauges connected to a digital readout. Grip strength was tested in three trials, and the greatest force was used for analysis; the values were represented as the ratio of strength in grams to body weight in grams. There are several ways to assess muscle quality, and we used the ratio of strength to muscle mass^10, 38^. We defined muscle quality as the ratio of grip strength in grams to triceps wet weight in milligrams.

### Tissue collection

All animals were deeply anesthetized using a 4.0–4.5% isoflurane gas with breathing medical air, maintained with 2.0% isoflurane during muscle collection, and then sacrificed at least 3 days after training sessions to exclude any short-term effects of exercise. The tissues were collected from triceps. The samples were quickly weighed, snap frozen with liquid nitrogen, and then stored at -80°C for mRNA analysis.

### RNA isolation and reverse transcription polymerase chain reaction (RT-PCR)

Transcript levels of target factors was assessed using RT-PCR as previously developed^39^. First, total RNA was extracted from triceps using a commercial TRI reagent, and 1 μg RNA was reverse transcribed into cDNA. The primer sequences for PCR were as follows: *IGF1* (fwd) 5′-TCCTTATGAATTGGCTTATC-3′, (rev) 5′-GTTTGTCATCTTCCATTCTGTT-3′; *AKT* (fwd) 5′-CGGCCACGCTACTTCCTCCTC-3′, (rev) 5′-GCCCATTCTTCCCGCTCCTCAG- 3′; *mTOR* (fwd) 5′-GCCCACGCCTGCCATACTTG- 3′, (rev) 5′-TCAGCTCCTCAGCTCC G G G T C T T C C T T G T T- 3 ′ ; *atrogin -1* ( fwd) 5′-CGTGCACGGCCAACAACC-3′, (rev) 5′-CCCGCCAACGTCTCCTCAAT- 3′; *MuRF1* (fwd) 5′-GGCTGCGAATCCCTACTGG- 3′, (rev) 5′-TGATCTTCTCGTCTTCGTGTTCCT- 3′. For each PCR assay, 18S was amplified with each target mRNA, yielding a target mRNA/18S ratio. The PCR products were separated by electrophoresis on 2% agarose gels and visualized by ethidium bromide for band sizes and product purity.

### Statistical analysis

All data are presented as means ± standard errors, with significance set at *p* < 0.05. The differences between groups were analyzed using one-way analysis of variance and post-hoc least significant difference comparisons. Statistical analysis was performed using Origin 8.0 and SPSS version 18.0 software.

## RESULTS

There were significant differences in changes in body weight and fat mass after 20 weeks of treatment among the groups (*p* < 0.001). After 20 weeks, body weight was significantly increased in the H (+57.31%, *p* < 0.001), H-RT (+41.66%, *p* < 0.001), and H-RTCN groups (+27.35%, *p* = 0.002) compared with that in the C group. However, there were no significant differences in body weight at 20 weeks between the C and H-CN groups. In addition, body weights after 20 weeks were significantly lower in the H-CN (-29%, *p* < 0.001) and H-RTCN groups (-19%, *p* = 0.002) compared with that in the H group. Fat mass at 20 weeks was also higher in the H (+91.68%, *p* < 0.001), H-RT (+103.15%, *p* < 0.001), and H-RTCN groups (+23.77%, *p* = 0.076) than in the C group. However, fat mass was significantly reduced in the H-CN (-42.63%, *p* < 0.001) and H-RTCN groups (-35.43%, *p* < 0.001) compared with that in the H group.

The weights of triceps did not significantly differ among groups (C: 106.34 ± 4.51 mg, H: 115.79 ± 14.07 mg, H-CN: 101.95 ± 4.77 mg, H-RT: 106.84 ± 5.61 mg, H-RTCN: 127.13 ± 6.53 mg; *p* = 0.069); however, normalizing the triceps weight to body weight yielded significant differences among groups (*p* < 0.001). Significantly decreased relative triceps weights were observed in the H (-31%, *p* < 0.001), H-CN (-13%, *p* = 0.042), and H-RT groups (-29%, *p* < 0.001) compared with that in the C group. Additionally, relative triceps weight was significantly higher in the H-CN (+27%, *p* = 0.007) and H-RTCN groups (+37%, *p* = 0.001) than in the H group.

Grip strength in the H group was significantly lower than in the C group (+76%, *p* < 0.001); in contrast, grip strength in the H-CN (+43%, *p* = 0.007), H-RT (+44%, *p* = 0.005), and H-RTCN groups (+73%, *p* < 0.001) was significantly greater than that in the H group. In particular, grip strength in the H-RTCN group was significantly greater than those in the H-CN (+20%, *p* = 0.03) and H-RT groups (+19%, *p* = 0.038). Muscle quality was significantly greater in the H-RT group than in the H (+33%, *p* = 0.013) and H-CN groups (+27%, *p* = 0.013), although there were no significant differences among groups (*p* = 0.066).

Although there were no significant differences in IGF1 mRNA expression among groups, Akt mRNA expression decreased in the H-CN (versus C: -22%, *p* = 0.022), H-RT (versus C: -34%, *p* = 0.001; versus H: -32%, *p* = 0.004), and H-RTCN groups (versus C: -31%, *p* = 0.003; versus H: -29%, *p* = 0.011). Additionally, mTOR mRNA expression increased in the H (+97%, *p* = 0.012), H-CN (+117%, *p* = 0.001), H-RT (+94%, *p* = 0.006), and H-RTCN groups (+104%, *p* = 0.005) compared with that in the C group, and atrogin1 expression was significantly decreased in the H-RTCN group compared with that in the H group (*p* = 0.006) and H-CN groups (*p* = 0.004) by 37% and 34%, respectively. MuRF1 expression was decreased in the H-RT (versus C: -34%, *p* = 0.011; versus H: 37%, *p* = 0.01) and H-RTCN groups (versus C: -66%, *p* < 0.001; versus H: -67%, *p* < 0.001; versus H-CN: -57%, *p* = 0.001; versus H-RT: -48%, *p* = 0.019). Interestingly, significant decreases in atrogin1 (-34%, *p* = 0.004) and MuRF1 expression (-57%, *p* = 0.001) were observed in the H-RTCN group compared with that in the H-CN group.

## DISCUSSION

HFD reduces muscle function and induces muscle atrophy and obesity^35-37, 40^. Regular CLA or n-3 treatment has been reported to reduce body fat mass in healthy adults, normal-weight healthy exercising adults, and overweight and obese adults^21-25, 29, 41-44^. However, many other studies have also reported that CLA or n-3 intake did not significantly affect body fat or lean body mass in lean or nonobese subjects and older adults^28, 45^. These different results indicate that the effects of CLA and n-3 supplements may vary with age, sex, obesity, physical activity level, and consumption duration. In the present study, we found that HFD for 20 weeks induced obesity (57.31% weight gain and 91.68% fat gain) and decreased skeletal muscle mass relative to body weight, whereas CLA plus n-3 supplementation with or without RT attenuated weight gain and skeletal muscle wasting. Furthermore, the 20-week HFD induced a reduction in muscle strength relative to body weight, whereas RT, CLA/n-3, and combined RT with CLA/n-3 prevented the decrease in muscle strength induced by HFD in middle-aged mice.

Muscle atrophy induced by an HFD is explained through various mechanisms, including an imbalance between protein synthesis and degradation. The ubiquitin proteasome system catalyzes the degradation of most cellular proteins^46^ and is one of the major pathways regulating muscle protein degradation; this system plays a central role in controlling muscle size^1^. Atrogin1 and MuRF1 are two E3 ubiquitin ligases that are important regulators of ubiquitin-mediated protein degradation in skeletal muscle. Although we did not find a significant increase in the expression of atrogin1 or MuRF1 after 20 weeks of HFD, these targets tended to show increased expression after consumption of the HFD. In fact, we found that RT with or without CLA/n-3 intake decreased *atrogin1* and *MuRF1* mRNA expression in skeletal muscle induced by the HFD in middle-aged mice. Moreover, the expression of atrophy-related genes was not significantly increased after long-term HFD. Interestingly, Sandri et al.^47^ reported that the isometric force of the gastrocnemius muscle and force per muscle weight was lower in atrogin1- and MuRF1-knockout mice at 15 and 26 months of age, respectively. These results indicated that the absence of the atrophy-related ubiquitin ligases atrogin1 and MuRF1 decreased force generation during isometric contraction. However, our results showed that RT with or without CLA/n-3 intake reduced muscle strength and quality, and these effects were exacerbated by consumption of the HFD, accompanied by decreases in atrogin1 and MuRF1 expression. The lack of *atrogin1* and *MuRF1* expression has been reported to prevent muscle atrophy in denervation mice3. In addition, atrogin1-knockdown mice^48^ and MuRF1-knockout mice^49^, which show increased muscle loss following fasting and dexamethasone administration, respectively, showed resistance to muscle atrophy. Taken together, these findings suggested that specific atrophy-related ubiquitin ligases may be expressed differently depending on the stage of the atrophy process and the model of muscle atrophy, e.g., immobilization, denervation, fasting, HFD, dexamethasone, or catabolic conditions. These genes may also be expressed differently depending on age, sex, and muscle type because these factors are known to affect muscle mass and strength. Various transcription factors, such as Smad3, p38 mitogen-activated protein kinase, and FoxO signaling, regulate the expression of *atrogin1* and *MuRF1* genes. In order to clarify differences in the expression of atrophy-related ubiquitin ligases, it will be necessary to analyze the various transcription factors together.

In our study, the expression of *mTOR* mRNA was significantly increased in the experimental groups compared with that in the control group consuming a normal diet; in contrast, differences in the expression of *IGF1* and *Akt* mRNA were not observed in HFD-induced obesity. Long-term CLA-n3 intake with or without RT was shown to decrease *Akt* expression and increase *mTOR* expression in mice fed an HFD. To date, the results of studies on the Akt/mTOR signaling pathway targeting animal models have been controversial. Despite the decrease in skeletal muscle mass due to aging^50-52^, Akt activation has been reported to be decreased^50, 53^, unchanged^51, 54^, or increased^52^. Skeletal muscle proteins consistently undergo metabolic turnover, muscle protein synthesis, and protein degradation, and the amount of protein is determined by the relative rate of protein synthesis and proteolysis. Changes in body composition due to metabolic conditions such as aging and obesity induce negative regulation of genes related to various signaling pathways and leading to an imbalance in protein synthesis/degradation in skeletal muscle. In order to clarify differences in expression of hypertrophy-related genes, it will be necessary to analyze the regenerative capacity of satellite cells, turnover of damaged mitochondria, and nuclear apoptosis simultaneously. Finally, this study had some limitations, making it difficult to compare directly with the results of previous studies, because we induced changes in body composition by long-term HFD, exercise intervention, and nutritional treatment together at the same time.

## CONCLUSION

HFD-mediated expression of genes involved in protein degradation was attenuated following 20-week RT with CLA/n-3 supplementation. Furthermore, CLA/n-3 with or without RT prevented weight gain, and RT with or without CLA/n-3 improved grip strength and muscle quality in middle-aged mice during HFD, albeit to a lesser degree for the latter. Therefore, RT with CLA/n-3 supplementation during HFD may improve muscle strength and quality by suppressing protein degradation. Future research is needed to verify these findings in middle-aged obese men and women.
